# Supplementary figures and images for: Transformer-based deep learning enables improved B-cell epitope prediction in parasitic pathogens: A proof-of-concept study on Fasciola hepatica
Source: PLoS Negl Trop Dis. 2025 Apr 29;19(4):e0012985. doi: 10.1371/journal.pntd.0012985 (PMC12064019; doi:10.1371/journal.pntd.0012985)

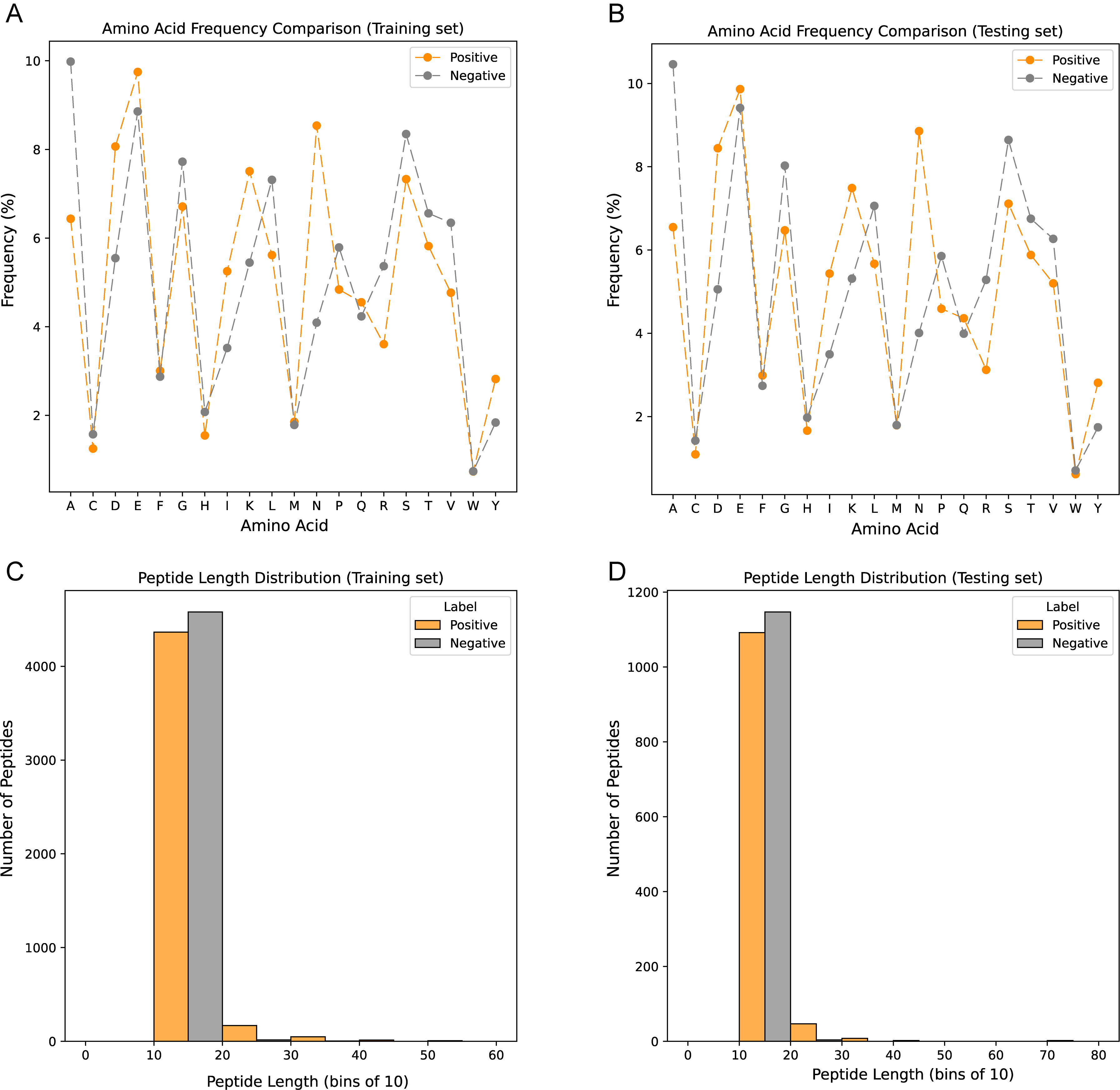

Supplement: S1 Fig — (A-B) Amino acid frequency in positive and negative samples from the training and testing datasets. (C-D) Peptide length distribution in positive and negative samples from the training and testing datasets. (TIF) [file pntd.0012985.s001.tif]

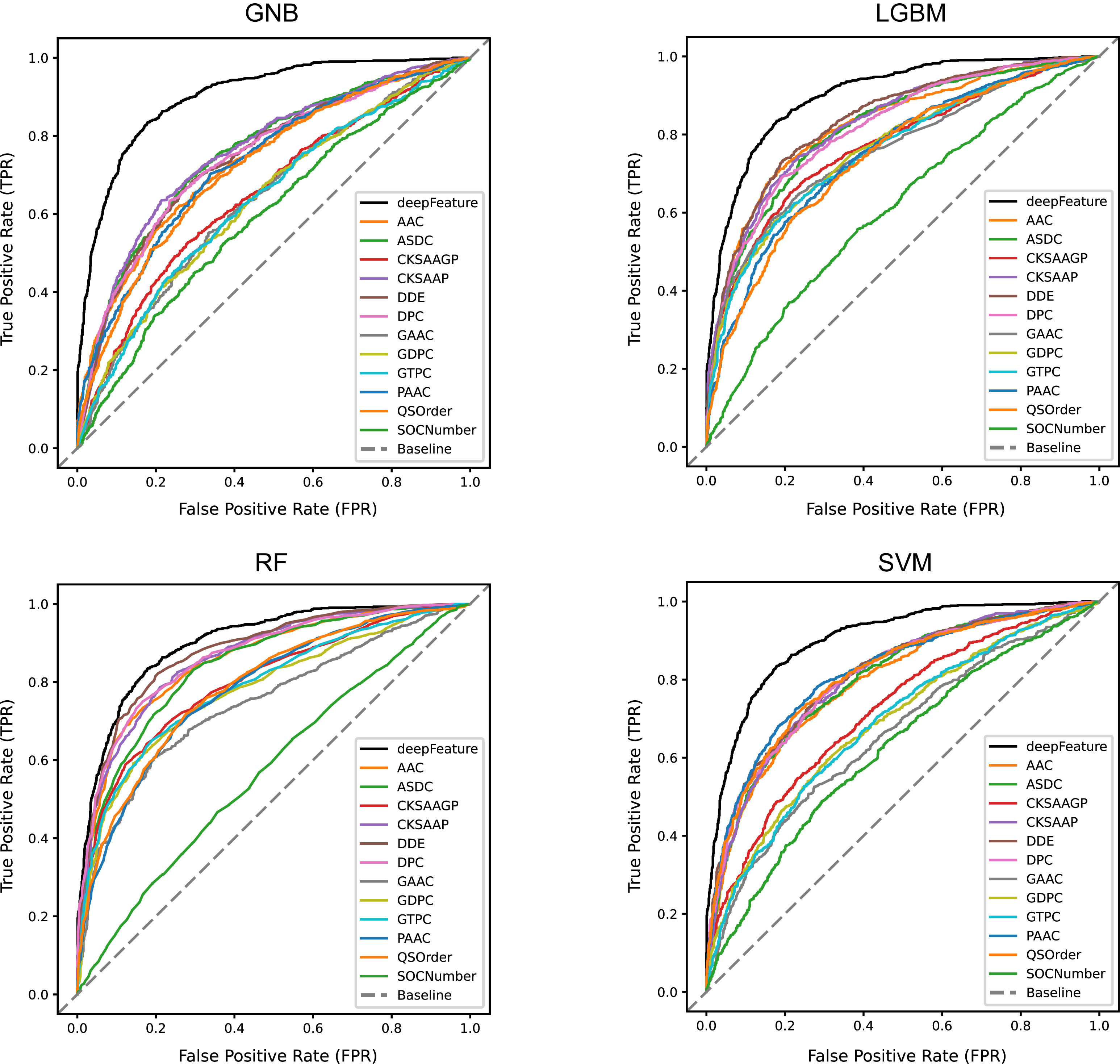

Supplement: S2 Fig — (TIF) [file pntd.0012985.s002.tif]

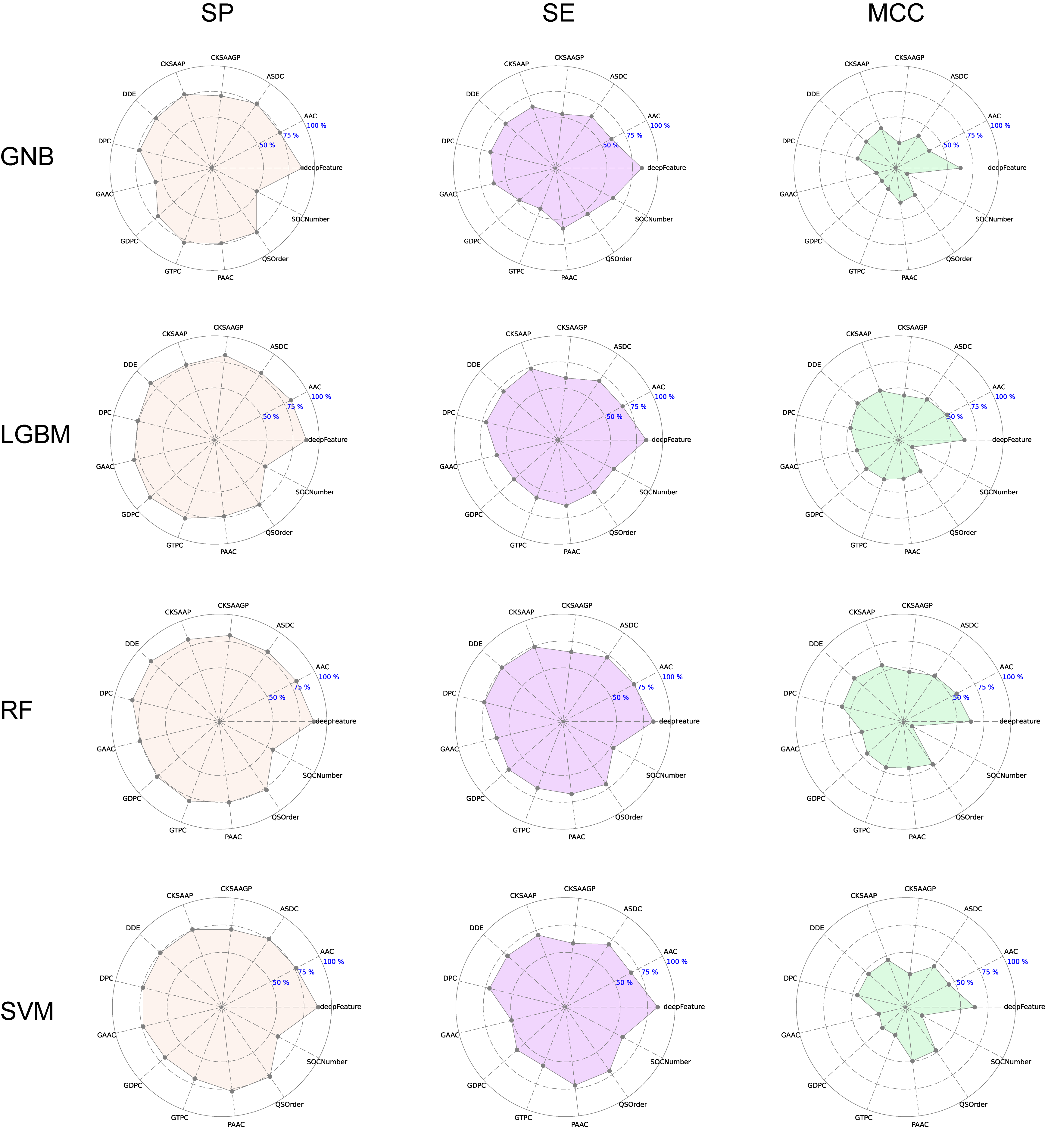

Supplement: S3 Fig — The chart highlights their respective performance in SP, SE, and MCC metrics. (TIF) [file pntd.0012985.s003.tif]
