# Supplementary material for: Transformer-based deep learning enables improved B-cell epitope prediction in parasitic pathogens: A proof-of-concept study on Fasciola hepatica
Source: PLoS Negl Trop Dis. 2025 Apr 29;19(4):e0012985. doi: 10.1371/journal.pntd.0012985 (PMC12064019; doi:10.1371/journal.pntd.0012985)
Supplement: Attachment — Submitted filename: reviewcomments.docx [file pntd.0012985.s008.docx]

The manuscript titled "Improved B-cell epitope identification in parasitic pathogens through a Transformer-driven model: Proof-of-concept in Fasciola hepatica" presents an innovative approach to predict B-cell epitopes (BCEs) using a Transformer-based deep learning model. The study is well-structured, methodologically sound, and provides compelling experimental validation using Fasciola hepatica as a case study. The findings have significant implications for epitope-based vaccine design, therapeutic antibody development, and diagnostics in parasitology. However, a few minor revisions are required to enhance clarity and address some methodological and presentation concerns.

Minor Comments:

1. Some sentences in the Introduction and Discussion sections are overly long and could be split for clarity.
2. The authors mentioned four traditional machine learning algorithms (GNB, SVM, RF and LGBM), while the GNB algorithms seems not be listed in line 122.
3. In line 241, the word “GNB” shorted for Gaussian Naïve Bayes seems correct. Please check the reset sections of the manuscript.
4. In line 296, the number ID behind “the LAP protein” seems Uniport ID, please add the Uniport ID before the number.
5. While the authors have compared their Transformer-based model with traditional machine learning approaches, additional discussion on how the Transformer architecture specifically enhances BCE prediction compared to sequence-based recurrent neural networks (RNNs) or attention-based models would be beneficial.
6. The introduction effectively sets up the importance of BCE prediction in parasitology. However, consider elaborating on the distinction between linear and conformational epitopes and their relative importance in vaccine design to provide more context for readers unfamiliar with the topic.
7. While the dataset preparation is described, please provide more details on the criteria for negative sample selection from IEDB. This will help clarify the robustness of the training dataset.
8. In Figure 2, the performance comparison charts for traditional machine learning models are informative, but the axis labels and legends could be made clearer for better readability.
9. In Figure 3, while the clustering heatmaps are valuable, they would benefit from more descriptive figure captions to guide the reader through the analysis.
10. In Figure 4, what is the meaning of pTM score? I think the author should explain them in the manuscript.
11. I think the authors used AF3 to model the vaccine molecules should be referenced in line 786.
12. In line 785, the word “as” is incorrect. Please check if the words are well-spelled.
