# Supplementary material for: Transformer-based deep learning enables improved B-cell epitope prediction in parasitic pathogens: A proof-of-concept study on Fasciola hepatica
Source: PLoS Negl Trop Dis. 2025 Apr 29;19(4):e0012985. doi: 10.1371/journal.pntd.0012985 (PMC12064019; doi:10.1371/journal.pntd.0012985)
Supplement: Attachment — Submitted filename: Comments.docx [file pntd.0012985.s009.docx]

Present work, titled as “*Improved B-cell epitope identification in parasitic pathogens through a Transformer-driven model: Proof-of-concept in Fasciola hepatica*”, is a demonstration of utilizing artificial intelligence tools to develop a deep learning model. The aim of this model (deepBCE-parasite) is to recognize B-cell epitopes (a group of amino acids present in parasitic proteins) which serve as targets for antibodies, thereby contributing towards facile development of therapeutic as well as diagnostic tools. Authors have creatively utilized the available resources (proteomics data, machine learning/artificial intelligence tools) to address immune-target study in parasitology.

Authors have chosen to study F. hepatica, a class of commonly observed parasitic flatworm, responsible for tropically neglected disease (fascioliasis). Transfer based deep learning model has been developed to predict B-cell epitopes from peptide sequences, in case of F. hepatica. This study presents the scope of utilizing artificial intelligence as a supporting tool in identifying/predicting therapeutic solutions to existing challenges. Authors have provided the background of current study and highlighted the limitations/challenges followed by desired future actions. The work can be accepted after addressing some minor concerns:

- Highlight the importance of LGBM algorithm along with supporting references, for better understanding of the readers.
- Authors should also discuss other limitations/challenges (in addition to rising drug-resistance) associated with the use of ‘conventional antiparasitic broad-spectrum drugs’, in the current scenario.
- Authors should highlight the possible advantages of incorporating artificial intelligence tools in such practices, to support or compliment experimental studies.
