# Supplementary material for: Transformer-based deep learning enables improved B-cell epitope prediction in parasitic pathogens: A proof-of-concept study on Fasciola hepatica
Source: PLoS Negl Trop Dis. 2025 Apr 29;19(4):e0012985. doi: 10.1371/journal.pntd.0012985 (PMC12064019; doi:10.1371/journal.pntd.0012985)
Supplement: Attachment — Submitted filename: PNTD-D-24-01725-Response to Reviewers.docx [file pntd.0012985.s010.docx]

Mar 5, 2025

Dr. Aysegul Taylan Ozkan

Academic Editor

*PLoS Neglected Tropical Diseases*

Dr. Jong-Yil Chai

Section Editor

*PLoS Neglected Tropical Diseases*

Dr. Shaden Kamhawi

co-Editor-in-Chief

*PLoS Neglected Tropical Diseases*

Dear Drs Ozkan, Chai and Kamhawi,

**Re: Revised Manuscript ID PNTD-D-24-01725.R1**

On behalf of all co-authors, I would like to thank you and the five anonymous reviewers very much for favorable comments and constructive suggestions on our manuscript (MS) ID **PNTD-D-24-01725**. The reviewers considered our MS to be of general interest to the readership of ***PLoS Neglected Tropical Diseases*** and recommended the acceptance of our MS for publication after revisions.

Therefore, we have revised the MS strictly according to the reviewers’ comments and suggestions. We used the “tracked changes” mode in the Microsoft Word to show the revised/changed text in the revised MS. Two MS files are uploaded: the “clean version” and the one showing “tracked changes”. In the following, we detail our point-by-point responses to the reviewers’ comments and suggestions. We made all our responses in blue for clarity.

We have done our best to address all comments and we sincerely hope that you find our MS revised to your satisfaction. We are looking forward to receiving your editorial decision soon and hope to see our work published in ***PLoS Neglected Tropical Diseases***.

With best wishes,

Rui

Rui-Si Hu, BVSc, MVSc, PhD in Veterinary Medicine | Postdoctoral Researcher in Computer Science (Bioinformatics), Distinguished Research Fellow

School of Health and Wellness Industry & School of Medicine, Sichuan University of Arts and Science, Dazhou, Sichuan Province 635000,

People’s Republic of China

Email: grishu0707@gmail.com

**Journal Requirements**

1) Please ensure that the CRediT author contributions listed for every co-author are completed accurately and in full.

At this stage, the following Authors/Authors require contributions: Rui-Si Hu, Kui Gu, Muhammad Ehsan, Sayed Haidar Abbas Raza, and Chun-Ren Wang. Please ensure that the full contributions of each author are acknowledged in the "Add/Edit/Remove Authors" section of our submission form. The list of CRediT author contributions may be found here: <https://journals.plos.org/plosntds/s/authorship#loc-author-contributions>.

**Response:** Thank you very much for your reminder. In the revised MS, we have updated and completed the CRediT author contributions for all mentioned authors in full accordance with the guidelines.

2) Please ensure that all Table files have corresponding citations and legends within the manuscript. Currently, Table 2 in your submission file inventory does not have an in-text citation. Please include the in-text citation of the table.

**Response:** Thank you very much for your reminder. The in-text citation for Table 2 has been added, and all tables have corresponding citations and legends.

3) Tables should not be uploaded as individual files. Please remove these files and include the Tables in your manuscript file as editable, cell-based objects. For more information about how to format tables, see our guidelines:

https://journals.plos.org/plosntds/s/tables

**Response:** Revised accordingly.

4) Some material included in your submission may be copyrighted. According to PLOSu2019s copyright policy, authors who use figures or other material (e.g., graphics, clipart, maps) from another author or copyright holder must demonstrate or obtain permission to publish this material under the Creative Commons Attribution 4.0 International (CC BY 4.0) License used by PLOS journals. Please closely review the details of PLOSu2019s copyright requirements here: PLOS Licenses and Copyright. If you need to request permissions from a copyright holder, you may use PLOS's Copyright Content Permission form.

Please respond directly to this email and provide any known details concerning your material's license terms and permissions required for reuse, even if you have not yet obtained copyright permissions or are unsure of your material's copyright compatibility. Once you have responded and addressed all other outstanding technical requirements, you may resubmit your manuscript within Editorial Manager.

Potential Copyright Issues:

i) Figures 1a, and 3a. Please confirm whether you drew the images / clip-art within the figure panels by hand. If you did not draw the images, please provide (a) a link to the source of the images or icons and their license / terms of use; or (b) written permission from the copyright holder to publish the images or icons under our CC BY 4.0 license. Alternatively, you may replace the images with open source alternatives. See these open source resources you may use to replace images / clip-art:

- https://commons.wikimedia.org - https://openclipart.org/.

**Response:** Thank you very much for your feedback. We confirm that Figure 1a was hand-drawn by us in the revised MS version, while Figure 3A’s parasite life cycle illustration of *Fasciola* was sourced from Servier Medical Art (https://smart.servier.com/), which provides images under a Creative Commons Attribution License (CC BY 4.0).

5) Please amend your detailed Financial Disclosure statement. This is published with the article. It must therefore be completed in full sentences and contain the exact wording you wish to be published.

1) State the initials, alongside each funding source, of each author to receive each grant. For example: "This work was supported by the National Institutes of Health (####### to AM; ###### to CJ) and the National Science Foundation (###### to AM)."

**Response:** Revised accordingly.

2) State what role the funders took in the study. If the funders had no role in your study, please state: "The funders had no role in study design, data collection and analysis, decision to publish, or preparation of the manuscript."

**Response:** Revised accordingly.

6) Your current Financial Disclosure states, "This work was supported by the National Natural Science Foundation of China [62202083]; the China Postdoctoral Science Foundation [2022M710618]; and the High-level Talent Research Start-up Project of Sichuan University of Arts and Science [WL085267]. ".

However, your funding information on the submission form indicates receiving one fund. Please ensure that the funders and grant numbers match between the Financial Disclosure field and the Funding Information tab in your submission form. Note that the funders must be provided in the same order in both places as well.

**Response:** Thank you very much for your feedback. I have reviewed and updated the funding information to ensure that the funders and grant numbers are consistent between the Financial Disclosure field and the Funding Information tab in the submission form.

Please indicate by return email the full and correct funding information for your study and confirm the order in which funding contributions should appear. Please be sure to indicate whether the funders played any role in the study design, data collection and analysis, decision to publish, or preparation of the manuscript.

**Response:** Thank you very much for your reminder. This work was supported by the following funding sources in the specified order: The National Natural Science Foundation of China (Grant No. 62202083 to RSH); The China Postdoctoral Science Foundation (Grant No. 2022M710618 to RSH); The High-level Talent Research Start-up Project of Sichuan University of Arts and Science (Grant No. 2024GCC16Z to RSH). The funders had no role in study design, data collection and analysis, decision to publish, or preparation of the manuscript.

7) Please amend the label of Figure S3 in the online submission form as it is currently uploaded as Figure S2.

**Response:** Thank you for pointing this out. The label of Figure S3 has been amended in the online submission form to correctly reflect its designation.

**Comments for the authors**

Please note that the reviews are uploaded as attachments.

Reviewers' Comments:

Reviewer's Responses to Questions

Key Review Criteria Required for Acceptance?

As you describe the new analyses required for acceptance, please consider the following:

*Methods*

-Are the objectives of the study clearly articulated with a clear testable hypothesis stated?

-Is the study design appropriate to address the stated objectives?

-Is the population clearly described and appropriate for the hypothesis being tested?

-Is the sample size sufficient to ensure adequate power to address the hypothesis being tested?

-Were correct statistical analysis used to support conclusions?

-Are there concerns about ethical or regulatory requirements being met?

*Results*

-Does the analysis presented match the analysis plan?

-Are the results clearly and completely presented?

-Are the figures (Tables, Images) of sufficient quality for clarity?

*Conclusions*

-Are the conclusions supported by the data presented?

-Are the limitations of analysis clearly described?

-Do the authors discuss how these data can be helpful to advance our understanding of the topic under study?

-Is public health relevance addressed?

**Response to the comments of Reviewer #1**

The manuscript titled "Improved B-cell epitope identification in parasitic pathogens through a Transformer-driven model: Proof-of-concept in *Fasciola hepatica*" presents an innovative approach to predict B-cell epitopes (BCEs) using a Transformer-based deep learning model. The study is well-structured, methodologically sound, and provides compelling experimental validation using *Fasciola hepatica* as a case study. The findings have significant implications for epitope-based vaccine design, therapeutic antibody development, and diagnostics in parasitology. However, a few minor revisions are required to enhance clarity and address some methodological and presentation concerns.

**Response:** We thank the Reviewer #1 very much for your positive feedback and constructive suggestions on our MS. We have carefully addressed all revisions to enhance the MS’s clarity and quality. All changes have been highlighted in the revised MS using “tracked changes”.

Minor Comments:

1. Some sentences in the Introduction and Discussion sections are overly long and could be split for clarity.

**Response:** In the revised MS, we have extensively optimized the sentence structures in the Introduction and Discussion sections.

2. The authors mentioned four traditional machine learning algorithms (GNB, SVM, RF and LGBM), while the GNB algorithms seems not be listed in line 122.

**Response:** We have added GNB algorithms in line 122.

3. In line 241, the word “GNB” shorted for Gaussian Naïve Bayes seems correct. Please check the reset sections of the manuscript.

**Response:** We have corrected the abbreviation.

4. In line 296, the number ID behind “the LAP protein” seems Uniport ID, please add the Uniport ID before the number.

**Response:** We have added the “Uniprot ID” before the LAP protein.

5. While the authors have compared their Transformer-based model with traditional machine learning approaches, additional discussion on how the Transformer architecture specifically enhances BCE prediction compared to sequence-based recurrent neural networks (RNNs) or attention-based models would be beneficial.

**Response:** Thank you very much for your constructive suggestions. In the revised MS, we have elaborated on how Transformers address the limitations of sequential processing and long-range dependency modeling in RNNs, while also highlighting their ability to efficiently extract hierarchical features through self-attention mechanisms. This additional discussion provides a clearer comparison of the strengths of Transformer-based approaches for BCE prediction.

6. The introduction effectively sets up the importance of BCE prediction in parasitology. However, consider elaborating on the distinction between linear and conformational epitopes and their relative importance in vaccine design to provide more context for readers unfamiliar with the topic.

**Response:** Thank you very much for your constructive suggestions. In the revised MS introduction part, we have clearly outlined the differences between linear and conformational epitopes.

7. While the dataset preparation is described, please provide more details on the criteria for negative sample selection from IEDB. This will help clarify the robustness of the training dataset.

**Response:** Thank you very much for your constructive suggestions. In the revised MS, we have provided more detailed description for negative dataset preparation. In fact, the IEDB provides an experimentally validated negative dataset of parasite BCEs, which can be extracted directly, and then deduplicated using CD-HIT and numerically balanced to match the positive sample set.

8. In Figure 2, the performance comparison charts for traditional machine learning models are informative, but the axis labels and legends could be made clearer for better readability.

**Response:** Thank you for your reminder. In the revised MS, we have updated the axes and legends in Figure 2, including the display of AUC values, to enhance its readability and clarity.

9. In Figure 3, while the clustering heatmaps are valuable, they would benefit from more descriptive figure captions to guide the reader through the analysis.

**Response:** Thank you for your reminder. In the revised MS, we have enhanced Figure 3 by incorporating more descriptive captions into the clustering heatmaps to facilitate a clearer guide for the reader throughout the analysis.

10. In Figure 4, what is the meaning of pTM score? I think the author should explain them in the manuscript.

**Response:** Thank you very much for your constructive suggestions. The pTM score provided by AlphaFold3, which serves as an indicator of protein structure prediction quality, is included in the legend; higher values denote greater model reliability. In the revised MS, we have added an explanation of pTM score in the legend of Figure 5 (original Figure 4).

11. I think the authors used AF3 to model the vaccine molecules should be referenced in line 786.

**Response:** Thank you for your reminder. In the revised MS, we have added the reference for AF3: “Abramson et al., 2024. Accurate structure prediction of biomolecular interactions with AlphaFold 3.”

12 In line 785, the word “as” is incorrect. Please check if the words are well-spelled.

**Response:** Revised accordingly.

Summary and General Comments

Minor revisions.

**Response to the comments of Reviewer #2**

Present work, titled as “Improved B-cell epitope identification in parasitic pathogens through a Transformer-driven model: Proof-of-concept in *Fasciola hepatica*”, is a demonstration of utilizing artificial intelligence tools to develop a deep learning model. The aim of this model (deepBCE-parasite) is to recognize B-cell epitopes (a group of amino acids present in parasitic proteins) which serve as targets for antibodies, thereby contributing towards facile development of therapeutic as well as diagnostic tools. Authors have creatively utilized the available resources (proteomics data, machine learning/artificial intelligence tools) to address immune-target study in parasitology.

Authors have chosen to study *F. hepatica*, a class of commonly observed parasitic flatworm, responsible for tropically neglected disease (fascioliasis). Transfer based deep learning model has been developed to predict B-cell epitopes from peptide sequences, in case of *F. hepatica*. This study presents the scope of utilizing artificial intelligence as a supporting tool in identifying/predicting therapeutic solutions to existing challenges. Authors have provided the background of current study and highlighted the limitations/challenges followed by desired future actions. The work can be accepted after addressing some minor concerns:

**Response:** We thank the Reviewer #2 very much for your positive feedback and constructive suggestions on our MS. We have carefully addressed all revisions to enhance the manuscript’s clarity and quality. All changes have been highlighted in the revised manuscript using “tracked changes”.

Highlight the importance of LGBM algorithm along with supporting references, for better understanding of the readers.

**Response:** Thank you for your reminder. In the revised MS, we have added the supporting references for the importance of LGBM algorithm. For example, feature selection.

Authors should also discuss other limitations/challenges (in addition to rising drug-resistance) associated with the use of ‘conventional antiparasitic broad-spectrum drugs’, in the current scenario.

**Response:** Thank you very much for your constructive suggestions. In addition to the rising issue of drug resistance, several other limitations and challenges are associated with the use of conventional antiparasitic broad-spectrum drugs, including toxicity and side effects, complex parasite life cycles, limited efficacy against chronic or latent infections, environmental persistence, and non-specific targeting. Among these, drug resistance remains one of the most significant challenges due to its global impact and the difficulty in reversing it. In the revised MS, we have expanded the context on these limitations and challenges, with a particular focus on the example of fascioliasis, highlighting how these issues affect current treatment strategies and emphasizing the need for alternative approaches.

Authors should highlight the possible advantages of incorporating artificial intelligence tools in such practices, to support or compliment experimental studies.

**Response:** Thank you very much for your valuable suggestions. We have highlighted the potential advantage of incorporating AI tools in B-cell epitope prediction in the revised MS. Specially, AI can efficiently process and analyze large-scale datasets, identify complex patterns, and make predictions in a shorter timeframe, thus complementing and enhancing traditional experimental studies. We believe that AI can not support but also improve the accuracy and efficiency of BCEs prediction. The discussion has been included in the revised MS to better illustrate the synergy between AI and experimental research.

Summary and General Comments

Manuscript is very well written.

**Response to the comments of Reviewer #3**

Hu et al., presents an innovative approach to B-cell epitope prediction using a Transformer-based deep learning model, specifically focusing on parasitic pathogens. While the study demonstrates strong technical merit and relevance to vaccine design, several issues require attention to improve the overall clarity, methodological rigor, and presentation. Below, I provide detailed comments and suggestions for each section of the manuscript.

**Response:** We thank the Reviewer #3 for constructive suggestions on our MS. We have carefully addressed all revisions to enhance the manuscript’s clarity and quality. All changes have been highlighted in the revised manuscript using “tracked changes”.

In the abstract part, the author provides a concise overview of the study, including the problem addressed, methodology, and results. However, it is overly technical and does not clearly articulate the novelty and significance of the research. For example, while metrics such as AUC are mentioned, their relevance is not explained. Additionally, the concluding statement lacks an impact-oriented summary of how the findings could contribute to vaccine design. I have some suggestions that require authors to clarify and revise them:

**Response:** We thank the Reviewer #3 very much for constructive suggestions. We have revised the abstract to address these points. The updated version now highlights the novelty and significance of the study, explains the relevance of the performance metrics, and provides a concise, impact-oriented conclusion.

First, please simplify the technical jargon to make it accessible to a broader audience. For instance, it perhaps more suitable for replacing "Transformer-driven model leveraging self-attention mechanisms" with "a novel deep learning model that improves prediction accuracy using advanced attention techniques.

**Response:** Thank you for your suggestions. In the revised MS, we have simplified the technical jargon to make it more accessible to a broader audience.

Second, clearly state the novelty of the approach, such as its ability to outperform traditional machine learning models in specific metrics.

**Response:** In the deep learning model deepBCE-Parasite, an accuracy of approximately 81% and an AUC of 0.90 were achieved in both 10-fold cross-validation and independent testing. However, in comparison to traditional machine learning models, we also evaluated 12 handcrafted features and four commonly used algorithms, finding that the best performance was achieved by the RF classifier using DDE features (Fig 2). The RF classifier demonstrated a training and independent test accuracy of approximately 80% and an AUC of 0.88. In contrast, the classification performance of RF was inferior to that of the deep learning model.

Lastly, can add a sentence on the broader implications of the study, e.g., "This study provides a robust tool for identifying B-cell epitopes, which could significantly enhance the development of vaccines for parasitic diseases."

**Response:** Thank you very much. We have adopted your suggestions.

The introduction provides a strong foundation on epitope prediction and underscores the importance of B-cell epitopes in vaccine development. However, the narrative is disrupted by an excessive focus on datasets and existing tools early on, and while the limitations of traditional methods are acknowledged, the specific research gap that this study addresses remains unclear. Reorganizing the introduction could significantly improve its clarity and flow. Thus, it is recommended to start with the significance of B-cell epitopes in vaccine design, followed by the challenges of existing prediction methods, such as limited accuracy and reliance on hand-crafted features. Moreover, clearly articulate the research gap and objective by stating, "To address these challenges, we developed a Transformer-based model to improve the accuracy and applicability of B-cell epitope prediction." Avoid redundancy, such as repeating the mention of the IEDB database, and briefly highlight the relevance of parasitic diseases like *Fasciola hepatica* to contextualize the study's focus. This approach will create a more engaging and logically structured introduction.

**Response:** Thank you very much for your valuable feedback. In the revised MS, we have refined the Introduction section by improving the logical flow and simplifying sentences to enhance clarity while eliminating redundancy. We believe these modifications improve the readability and coherence of the MS.

The Methods section provides a comprehensive explanation of the Transformer architecture and methodology, but its technical density may overwhelm non-technical readers. For readers without a deep learning background, in order to improve accessibility and clarity, simplify the explanation of the Transformer architecture and consider including a flowchart or visual chart to illustrate key components such as attention layers and embeddings.

**Response:** In the revised MS, we have simplified the explanation of the Transformer architecture to make it more accessible to non-technical readers, using intuitive analogies and avoiding overly technical jargon. Additionally, we have a Figure 1 to include a clear flowchart that visually illustrates the key components of the Transformer, such as the encoder-decoder structure, attention layers, embeddings, and classification. These changes aim to improve clarity and accessibility while maintaining the necessary technical depth for expert readers.

Also, please expand on the data preprocessing steps by describing how the dataset was balanced (e.g., oversampling) and detailing the processes for removing duplicates or noise.

**Response:** Thank you very much for your insightful comments. In this study, we used a balanced dataset consisting of positive and negative samples. Therefore, we did not apply oversampling or similar techniques. Utilizing balanced data is a common approach in both deep learning and machine learning, as it helps to mitigate overfitting issues caused by data imbalance. Furthermore, the balance of BCEs data for parasitic pathogens is inherently difficult to estimate. To enhance model training, we performed data preprocessing using CD-HIT to remove noise from both positive and negative samples, thereby improving the overall data quality and the robustness of our model.

Provide justification for the chosen hyperparameters, such as stating, "The choice of two encoder layers and eight attention heads was informed by prior studies and optimization experiments." Additionally, clarify the validation strategy by explicitly mentioning whether k-fold CV or an independent test set was used, and discuss measures taken to prevent overfitting. Finally, consider rephrasing overly technical sentences for broader readability. For instance, revise "The model utilized self-attention mechanisms to capture sequence dependencies" to "The model's attention mechanisms effectively identified patterns within the sequence data." These changes will make the section more engaging and accessible while maintaining its technical rigor.

**Response:** The choice of two encoder layers and eight attention heads was informed by our optimization experiments, ensuring a balance between model complexity and computational efficiency. These hyperparameters were customizable, and this configuration performed best in our comparisons. We used **10-fold cross-validation** and **an independent test set**, with no signs of overfitting, demonstrating the model’s robustness and generalizability. Moreover, we have revised the MS to improve readability by rephrasing overly technical sentences while maintaining scientific rigor. We appreciate your constructive suggestions, which have significantly enhanced the clarity and quality of our work.

In the Results section, while performance metrics such as AUC and MCC are provided, their significance in the context of vaccine design is not clearly articulated.

**Response:** In machine learning and deep learning model training, performance metrics such as AUC and MCC are crucial for evaluating model effectiveness, which is why they are widely used. The goal of our study was to train models that could accurately predict BCEs from parasitic pathogens. The predicted BCEs provide a scientific basis for identifying potential vaccine and diagnostic tool targets. Additionally, we used *Fasciola hepatica* as a case study, which further validated the model’s effectiveness.

For example, why does achieving an AUC of 0.90 matter, and how does it advance the field? Similarly, although the figures and tables are informative, do the captions provide enough detail to ensure clarity and proper interpretation for the reader?

**Response:** Thank you for your valuable feedback. AUC of 0.90 is capable of highlighting the model’s strong predictive ability in machine learning, suggesting it is powerful for identifying BCEs. We have also revised the figure and table captions according to your and other reviewers’ suggestions, in order to ensure clarity and ease of interpretation. We appreciate your insights, which have helped improve the quality of our MS.

To enhance this section, qualitative examples would help demonstrate the model's real-world utility. For instance, highlighting specific epitopes that the Transformer model successfully identified but traditional methods missed would strengthen the narrative. A brief case study showing the model’s practical application could further illustrate its value.

**Response:** Thank you for your constructive suggestions. In response, we have highlighted specific BCEs identified by our Transformer model that were missed by traditional methods, demonstrating its enhanced predictive power. Additionally, we have included a brief case study using *Fasciola hepatica* to illustrate the model’s practical application, further emphasizing its real-world utility.

Moreover, explaining why the Transformer outperforms traditional methods like SVM or Random Forest would add depth to the discussion—what aspects of the model contribute to its superior performance?

**Response:** Thank you for your valuable feedback and constructive suggestions. The evaluation of models depends on the performance metrics such as AUC and MCC, which were commonly used in machine learning and deep learning. In this study, deepBCE-Parasite achieved an AUC of 0.90 and 81% accuracy, outperforming traditional models like RF or SVM, which reached an AUC of 0.88. The Transformer model’s advantage lies in its self-attention mechanism, which captures long-range dependencies, enhancing feature learning over methods.

Finally, acknowledging any limitations of the model, such as specific types of epitopes it struggles to predict, or potential dataset biases, would provide a more balanced perspective.

**Response:** We acknowledge that our model has certain limitations, particularly given its accuracy of 81%, which leaves some room for improvement. Future studies could enhance performance by incorporating larger and higher-quality datasets to further optimize the model. Additionally, exploring more state-of-the-art deep learning frameworks may help refine predictions and improve generalizability.

Other comments for experiment validation

Why did the authors choose *Fasciola hepatica* as the research subject to validate the effectiveness of the model? Also, the authors highlighted four proteins, namely Glutathione transferase, Leucine aminopeptidase, Annexin, and *β*-Actin, to present the results. However, in the context of *Fasciola hepatica* research, these proteins have not demonstrated particularly ideal immune protection in livestock animals, even though they may exhibit some immunogenic effects. Additionally, can the deep learning and machine learning models developed in this study be applied to research on other parasites? And explain how this method could be applied to other parasitic pathogens or even other classes of pathogens (e.g., viral or bacterial epitopes)?

**Response:** Thank you for your thoughtful comments and insightful questions. We chose *Fasciola hepatica* as the research subject because it is a well-studied parasitic pathogen within our research team, and we have access to relevant experimental samples. This allowed us to effectively validate the model’s predictions using real-world data. Regarding the selection of Glutathione transferase, Leucine aminopeptidase, Annexin, and *β*-Actin, these proteins were chosen based on systematic summaries from prior review study (Toet et al., 2014; doi: 10.1016/j.ijpara.2014.07.011). Additionally, they have been evaluated for immunogenicity in previous animal experiments, showing some level of immune response upon vaccination. While they may not provide ideal immune protection in livestock, their documented immunogenicity makes them suitable for validating our model’s predictive capability in identifying BCEs. In terms of model applicability, deep learning and machine learning approaches rely heavily on data volume. A model trained on BCE data from a single parasite would be insufficiently generalized. Our models were trained using a diverse dataset, enabling its application to a wide range of human and animal parasites. However, its performance on non-parasitic pathogens (such as viral or bacterial epitopes) may be suboptimal, as the training data did not include such pathogens. To extend its applicability beyond parasites, the model would require retraining with a broader dataset that incorporates BCEs from different pathogen classes.

(6) The overall writing and logic are smooth, but the content and spelling throughout the text need careful checking. For example, on line 241, the abbreviation for Gaussian Naive Bayes should be GNB.

**Response:** Thank you for your positive comments. The abbreviation for GNB has been revised accordingly.

**Response to the comments of Reviewer #4**

This manuscript by Hu et al describes the development of a deep learning model, deepBCE-Parasite, which is applied in the identification of linear B-cell epitopes in parasitic pathogens, with a focus on *Fasciola hepatica*, a parasite of major health and economic importance. The authors have applied the Transformer architecture in designing a model that predicts BCEs from amino acid sequences using state-of-the-art self-attention mechanisms. In contrast, the deep learning approach significantly outperforms traditional machine learning methods such as Support Vector Machines and Random Forests with up to an accuracy of 81%, though robust performance across testing metrics is shown. The authors were able to use proteomic data from *F. hepatica* and predict eight BCEs derived from the leucine aminopeptidase protein, a known vaccine candidate, as a form of demonstration of its application. Experimental confirmation using dot-blot assays has been done on these, which shows the proof of concept that deep learning can help speed up BCE identification by reducing dependency on expensive and time-consuming experimental methods. It would be helpful in the development of vaccines, therapeutic antibody design, and diagnostics against neglected tropical diseases. In their paper, focusing on linear epitopes, the authors feel their future work needs to be directed towards conformational epitopes for better biological applicability. This manuscript is well written and I have a few suggestions for the improvement of the manuscript.

**Response:** We thank the Reviewer #4 very much for your positive feedback and constructive suggestions on our MS. We have carefully addressed all revisions to enhance the MS’s clarity and quality. All changes have been highlighted in the revised manuscript using “tracked changes”.

1. While the authors tested the model on *Fasciola hepatica*, its utility across other parasitic species was not thoroughly explored. The authors could test the model on other protozoan and helminthic parasites to demonstrate its generalizability and robustness in predicting BCEs for a wider range of pathogens.

**Response:** Thank you for raising this important point regarding the generalizability of our model. We agree that demonstrating the utility of our approach across diverse parasitic species would significantly strengthen its broader applicability. However, in the current study, our primary focus was to establish a proof-of-concept framework using *Fasciola hepatica* as a case study, given its well-characterized importance in veterinary and human parasitology and the availability of high-quality proteomic datasets required for experimental validation.

2. Experimental validation was limited to eight peptides from a single protein. This small sample size may not fully reflect the model's predictive accuracy or its applicability to diverse proteomes. The authors should acknowledge this limitation.

**Response:** We acknowledge that our experimental validation was limited to eight peptides from a single protein. We agree that this relatively small sample size may not fully represent the model’s performance across diverse proteomes. As noted in the revised MS, we have added a discussion of this limitation and emphasized the need for future studies to evaluate our model on a broader range of proteins and larger datasets.

3. While the model outperformed traditional machine learning approaches, it was not compared to state-of-the-art epitope prediction tools like DiscoTope or BepiPred-3.0. The authors could benchmark deepBCE-Parasite against other advanced models to establish its competitive edge and address any potential limitations in its methodology.

**Response:** Thank you very much for your constructive suggestions. In the revised MS, we have benchmarked our deepBCE and BCE_RF models on new independent test sets, while also evaluating existing state-of-the-art linear BCE models, including EpiDope and BepiPred3, which were trained on extensive BCE datasets, not specific to parasite datasets. The benchmark results have been displayed in the newly generated **Figure 3** and **Table 3**. The performance evaluation demonstrates that the deepBCE and BCE_RF models, trained on parasite-specific datasets, consistently outperformed EpiDope and BepiPred3 across various evaluation metrics, underscoring their enhanced adaptability and efficacy in parasite-related BCE prediction tasks. Through this comparison, we can clearly observe that models trained on non-parasite data exhibit inferior performance when benchmarked on parasite-specific test sets. Regarding your mention of DiscoTope, it is a tool designed for conformational epitope prediction and therefore cannot be directly compared in this context.

4. The authors should clearly state the limitations of their study.

**Response:** Thank you for your suggestions. In the revised MS, we have clearly stated the limitations of our study. For instance, we have not addressed the prediction of conformational epitopes, and the performance of the predictive model is not yet sufficiently high. This may be closely related to data processing and the volume of data. There is still big room for improvement in the model.

Yes, the analysis aligns with the outlined plan. The manuscript describes the development and validation of the deepBCE-Parasite model.

Yes, the results are generally well-organized and clearly presented

Yes, the figures are of good quality.

Yes, the conclusions are supported by the data.

The authors acknowledge some limitations, such as the model's focus on linear epitopes and the lack of conformational epitope prediction, which reduces its applicability to real-world antigen-antibody interactions. However, they do not fully discuss other potential shortcomings, such as dataset bias, limited validation across diverse parasites, or the relatively small scope of experimental validation.

Yes. The authors emphasize the significance of their model in advancing epitope-based vaccine design, antibody therapeutics, and diagnostics for parasitic diseases.

Yes. The authors address the public health implications of their work, noting that *Fasciola hepatica* is a globally significant zoonotic parasite and emphasizing the potential of the model to aid in vaccine development and diagnostic tools for neglected tropical diseases.

**Response:** We thank the Reviewer #4 again for your positive feedback and constructive suggestions on our MS.

Summary and General Comments

Minor Revision

**Response to the comments of Reviewer #5**

This article comprehensively addresses the development of both deep learning and traditional machine learning models for predicting linear B cell epitopes (BCEs) in parasitic organisms, focusing on protozoa and helminths. The manuscript successfully demonstrates the benefits of Transformer-based architectures (in particular, its encoder-decoder approach with multi-head attention) over traditional methods, while providing an insightful case study on *Fasciola hepatica*. The validation of leucine aminopeptidase (LAP)-derived epitopes in the lab lends additional credence to the computational predictions.

**Response:** We thank the Reviewer #5 very much for your positive feedback and constructive suggestions on our MS. We have carefully addressed all revisions to enhance the manuscript’s clarity and quality. All changes have been highlighted in the revised manuscript using “tracked changes”.

1.Some details—particularly hyperparameter choices and rationale—could be expanded for reproducibility. For instance, justify why certain embedding dimensions or numbers of heads were selected.

Comparison Across Multiple Models

**Response:** Thank you for your valuable suggestions. In the revised MS, we have added detailed descriptions of the key hyperparameters and their rationale. In our study, for example, we chose dimension embedding (dk=256) to balance model capacity and computational efficiency. This dimension allows the model to capture rich semantic relationships between amino acids without excessive memory usage or training time. We used 8 attention heads to enable the model to focus on diverse aspects of the input sequence simultaneously, capturing both local and global dependencies effectively. These hyperparameters were carefully tuned through extensive experimentation and cross-validation comparison across multiple models to achieve the best performance on our dataset. Also, we have included a comprehensive summary of all parameters in S1 Table for reproducibility.

2. Add more discussion on how these findings could accelerate or integrate into wider vaccine and diagnostic test pipelines in veterinary or human parasitology.

**Response:** Thank you very much for your constructive suggestions. Our findings have significant potential to accelerate and integrate into vaccine development and diagnostic test pipelines in both veterinary and human parasitology. In particular, integrating omics datasets (e.g., proteomics) would enhance the identification of vaccine targets and diagnostic markers. In the revised MS, we have expanded the discussion on the utility of combining our approach with proteomics data to improve the accuracy and applicability of our predictions.

3. Increase the clarity around experimental methods (e.g., how peptides were chosen for validation and how many repetitions were performed). If possible, include quantification (intensity measurements) in a table or figure to show how signal intensities compare across peptides.

**Response:** Thank you very much for your constructive suggestions. In this study, we selected 8 peptide sequences derived from the LAP protein as validation targets based on the predicted antigenicity from our developed deep learning and machine learning models. These peptides were experimentally validated via dot-blot assays, with **three independent repetitions** to ensure reproducibility. Of these, 7 peptides showed positive reactivity with *Fasciola hepatica*-positive sera, confirming their specific binding to antibodies (Fig. 6B). Regarding the comment on signal intensity quantification, we acknowledge that such analysis could provide additional insights. However, the primary goal of this study was to confirm the presence or absence of reactivity between the peptides and specific antibodies, rather than to quantify signal intensity. Nevertheless, we appreciate this valuable suggestion and will consider incorporating quantitative intensity measurements in future studies to further enhance the robustness of our findings.

4. Provide a clearer roadmap of how the proposed pipeline can be extended to 3D data and conformational epitopes in future work. A brief discussion on potential synergy with AlphaFold or related structural modeling tools would be beneficial.

**Response:** The primary goal of this study was to develop a predictive model for linear B-cell epitopes. However, we fully acknowledge the importance of extending this pipeline to 3D data and conformational epitopes in future work. In the revised MS, we have added a discussion on how our approach can be integrated with advanced structural modeling tools, such as AlphaFold, to improve the prediction of conformational epitopes in parasitic proteins.

Reiterate or cross-reference the original analysis plan in the Results section to show how each step (e.g., cross-validation, feature selection, benchmarking) was carried out exactly as proposed.

Strengthen the connection to public health by explicitly mentioning how more accurate BCE prediction can reduce infection rates, improve diagnostics, or inform surveillance programs.

**Response:** Thank you very much for your constructive suggestions. In the revised MS, we have strengthened the connection to public health by highlighting how more accurate B-cell epitope prediction can reduce infection rates through targeted vaccine design, improve diagnostics by enabling the development of highly specific tests, and inform surveillance programs by identifying conserved epitopes across emerging pathogen strains. These advancements can significantly enhance disease prevention, early detection, and outbreak containment, particularly in endemic and low-resource settings.

Summary and General Comments

Overall, the study’s design, methodology, and results are presented cohesively. Once the minor revisions—particularly regarding clarity on experimental methods, dataset annotation, and limitations—are addressed, the manuscript will be suitable for publication. The revised paper would serve as a valuable resource for researchers working on vaccine design and diagnostic test development in parasitology and related fields.

**Response:** We thank the Reviewer #5 again for your positive feedback and constructive suggestions on our MS.
